# miR-101-3p Serves as a Tumor Suppressor for Renal Cell Carcinoma and Inhibits Its Invasion and Metastasis by Targeting EZH2

**DOI:** 10.1155/2021/9950749

**Published:** 2021-07-07

**Authors:** Yunze Dong, Yuchen Gao, Tiancheng Xie, Huan Liu, Xiangcheng Zhan, Yunfei Xu

**Affiliations:** Department of Urology, Shanghai Tenth People's Hospital, School of Medicine in Tongji University, 301 Yanchang Road, Jing'an District, Shanghai 200072, China

## Abstract

**Background:**

The role of miRNAs in renal cell carcinoma (RCC) is not certain. We wanted to study the biological functions and potential mechanisms of miR-101-3p in RCC.

**Methods:**

miR-101-3p was inhibited in A498 and OSRC-2 (two RCC cell lines). We studied its effect on cell invasion and proliferation. Target EZH2 of miR-101-3p was designated by different methods, including luciferase functional analysis and Western blotting. The expression level of the target gene in treated cells was quantitatively analyzed by quantitative real-time polymerase chain reaction. In addition, induction of miR-101-3p to prevent tumor formation of A498 cells in mice was further studied.

**Results:**

The overexpression of miR-101-3p significantly inhibited the proliferation, migration, and invasion in two RCC cells. Western blotting and luciferase functional analysis indicated that miR-101-3p regulated the expression of EZH2 in two cell lines. Mice inoculated with A498 and OSRC-2 cells transfected with miR-101-3p mimics showed significantly smaller xenografts and weaker EZH2 expression levels than the control group.

**Conclusions:**

miR-101-3p inhibited RCC cell proliferation, migration, and invasion by targeting EZH2.

## 1. Introduction

RCC is one of the most common malignancies of the urinary system [1], accounting for about 2%~3% of adult malignancies [2]. For localized and early-stage RCC, the best treatment is radical and partial nephrectomy, which has a 92.6% 5-year survival rate [3]. However, for metastatic RCC, the 5-year survival rate is expected to be very poor due to its resistance to radiotherapy and chemotherapy [4]. Although the advent of antiangiogenic drugs with tyrosine kinase inhibitors (TKI) has improved the prognosis of RCC, the associated resistance remains elusive [5]. It is important to uncover the mechanism of renal cancer and find potential prognostic biomarkers and therapeutic targets for renal cancer. Thus, we sought to find the molecular mechanism underlying RCC pathogenesis.

miRNA is a small nontranslated RNA that inhibits the expression of target genes through translation inhibition or transcriptional silencing [6]. Current studies have shown that miRNAs can act on at least 30% of protein-coding genes [7]. With the continuous development of technology, various miRNAs have been identified and have been widely proven to play an important role in tumorigenesis. Among them, miR-101-3p has been declared to regulate cancer development by acting as a tumor suppressor or an oncogene in tumorigenesis. For example, Wang et al. found that miR-101-3p promotes apoptosis of oral cancer cells by targeting BICC1 [8]. Wu et al. reported that it diametrically targets SRF to inhibit HOX transcript antisense RNA- (HOTAIR-) induced proliferation of gastric cancer cells [9]. In hepatocellular carcinoma, the LINC00052/miR-101-3p axis inhibited cell proliferation and metastasis by targeting SOX9 [10]. In non-small-cell lung cancer (NSCLC), it inhibits the growth and metastasis of NSCLC by blocking the PI3K/Akt signaling pathway by targeting MALAT-1 [11]. However, until now, its roles and underlying molecular mechanisms in RCC progression still remain unknown and need to be elucidated.

Thus, we focused on the role of miR-101-3p in RCC and found its low expression in RCC tissue based on the starBase database and clinical tumor specimen analysis. Its overexpression inhibited the proliferation, invasion, and migration of RCC cells. In addition, we found that the enhancer of zeste 2 polycomb repressive complex 2 subunit (EZH2) is a direct downstream target of miR-101-3p. Then, this miRNA in RCC was studied using A498 and OSRC-2 cells as models. These two cell lines have been widely used in the study of RCC. Therefore, we used the functional gain/loss method to demonstrate that it affected the proliferation and invasion of RCC cells in vitro and in vivo, respectively. Our findings suggest the oncogenic role of miR-101-3p in RCC and may provide a new potential target for RCC therapy.

## 2. Materials and Methods

### 2.1. RCC Tissue Samples

Between January 2019 and June 2020, 11 RCC samples and normal samples were collected from Shanghai Tenth People's Hospital. Fresh tumor tissue was stored in liquid nitrogen to avoid RNA degradation. The specimens were used with the consent of all patients and approved by the Ethics Committee of Shanghai Tenth People's Hospital of Tongji University.

All procedures in this study were performed in accordance with the ethical standards of the institutional and/or national research committee and with the World Medical Association's Declaration of Helsinki.

### 2.2. Bioinformatics Analysis

The GEO dataset GSE16441 was used for analysis. In this dataset, microRNAs from 17 RCC tumors and 17 corresponding nontumor samples were hybridized on a single-channel platform for miRNA expression analysis. Differentially expressed miRNAs were identified and presented by a clustered heat map and volcano plot [12, 13]. The expression of miR-101-3p and EZH2 was evaluated based on the starBase database (http://starbase.sysu.edu.cn/). The potential target genes of miR-101-3p were predicted using TargetScan (http://www.targetscan.org/), miRTarBase (http://mirtarbase.cuhk.edu.cn/), and miRDB (http://mirdb.org/) [14].

### 2.3. Cell Culture

Human RCC cell lines A498, OSRC-2, 786-O, and SW839 and human normal kidney tubular epithelial cell line (HK-2) were purchased from the Cell Bank of the Chinese Academy of Sciences (Shanghai, China) and were cultured, respectively, in DMEM (Invitrogen, Carlsbad, CA) supplemented with 10% fetal bovine serum (FBS), 50 U/ml of penicillin, and 50 *μ*g/ml of streptomycin (Invitrogen).

### 2.4. RNA Extraction, Reverse Transcription, and Quantitative Real-Time PCR (qRT-PCR) Analyses

Total RNA was measured using a rapid RNA extraction kit (Epizyme, Shanghai). The purity and concentration of RNA were measured using a NanoDrop 2000 spectrophotometer. The cDNA was synthesized using a Bulge Loop™ miRNA RT primer kit (RiboBio, China). An SYBR green qPCR kit (Yeason, Guangzhou, China) was used for qRT-PCR. Expressions of miR-101-3p and EZH2 were normalized to GAPDH or U6 applying the 2-*ΔΔ*Ct method.

### 2.5. Western Blotting Analysis

The sample is lysed on ice for 30 minutes with a RIPA buffer (1 : 100 protease inhibitors). After measuring the protein content, calculate the sample amount. After adding enough electrophoresis solution, start to prepare for loading. After electrophoresis, the electrophoresis can be stopped just after the bromophenol blue runs out, and the membrane is transferred. The hybridization was carried out overnight at 4°C (1 : 10000 for GAPDH, 1 : 1000 for EZH2). Then, it was extensively washed and incubated in a blocking buffer for 1 h at room temperature with appropriate horseradish peroxidase combined with a secondary antibody (1 : 1000). After three times of washing and film scanning, we analyzed the molecular weight and net optical density value of the target band with the gel image processing system.

### 2.6. Cell Proliferation Assays

Cell Counting Kit-8 is used to measure cell proliferation. Briefly, 1 × 10^3^ transfected RCC cells per well were seeded into 96-well plates and cultured for 24-120 h. Every 24 h, we measured optical density values using an automatic microplate reader (BioTek Solutions, Thousand Oaks, CA, USA) and incubated with CCK-8 (10 *μ*l) in 5% CO_2_ for 2 h [15].

### 2.7. Wound Healing Assays

First, draw horizontal lines evenly behind the 6-hole plate with a marker, cross the hole every 1 cm, and then lay the plate. On the next day, lines perpendicular to the horizontal lines were scratched with a spearhead. Then, wash the cells with PBS 3 times, and the cells were removed. After adding a serum-free medium, the cells were cultured in an incubator at 37°C and 5% CO_2_. Samples were taken at 0, 6, 12, and 24 h, and photos were taken.

### 2.8. Transwell Assays

Dilute Matrigel gel with a serum-free cell culture medium or PBS buffer at the ratio of 1 : 8 at 4°C, take 100 *μ*l and evenly smear on the surface of the polycarbonate membrane in the upper chamber, place at 37°C for 0.5-1 h, and polymerize it into a gel. Add a medium containing 10% FBS or chemokines to the lower chamber of the 6-well plate, take cell suspension to the upper chamber, and finally put it into the incubator for 12-48 h culture, cell fixation, cell staining, and counting.

### 2.9. Cell Transfection

The miRNA negative control (miR-NC), miR-101-3p mimics, and inhibitors were transfected instantaneously using Lipofectamine 2000 according to the manufacturer's protocol. In the overexpressed EZH2, the EZH2 vector and the overexpressed plasmid (oeEZH2) were synthesized by IBSBIO Biotechnology. We transfected the plasmid into HEK293T cells and packaged lentivirus. After 24 h, the lentivirus supernatant was collected and used to infect cells.

### 2.10. Luciferase Reporter Assays

The binding sites of miR-101-3p and EZH2 were obtained from TargetScan, and vectors containing the mutant-type (Mut) or wild-type (WT) sequence were synthesized by Genomeditech (China). Cultivate HEK293T cells, inoculate them in 24-well plates, and grow them for 10-24 hours. The reporter gene plasmid and transcription factor expression plasmid are cotransfected into cells. The protein is extracted and used for luciferase detection. Add the substrate and measure the luciferase activity. Calculate relative fluorescence intensity and compare with the no-load control.

### 2.11. Immunohistochemistry (IHC)

IHC was performed to assess the expression level of EZH2 in the tissues, as described earlier [16]. Frozen sections 4-8 *μ*m were placed at room temperature for 30 minutes, fixed in acetone at 4°C for 10 minutes, washed with PBS for 5 minutes 3x, and incubated with hydrogen peroxide for 5-10 minutes to eliminate endogenous peroxidase activity.

### 2.12. Statistical Analysis

Statistical analysis was performed using GraphPad Prism 7 (GraphPad Software, CA). All data were mean ± standard deviation (SD) of three independent experiments. Statistical significance between groups was analyzed using Student's *t*-test [17, 18]. Correlations between miR-101-3p and EZH2 were tested using Pearson's correlation coefficient analysis. All data were examined and statistically significant at *P* < 0.05 [19].

## 3. Results

### 3.1. miR-101-3p Was Upregulated in RCC Tissues and Cell Lines

**Figure 1:**
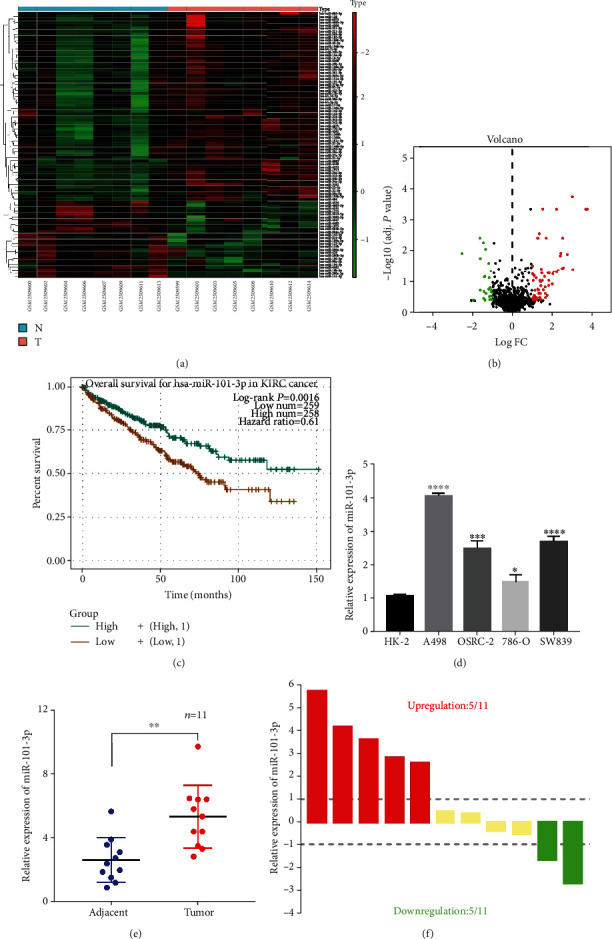
miR-101-3p was upregulated in RCC tissues and cell lines. (a, b) Expression level of miR-454 in the miRNA expression profiling array was presented by the clustered heat map and volcano plot. (c) Kaplan-Meier survival analyses indicated that high miR-101-3p expression was closely related to poor survival. (d) miR-101-3p was highly expressed in RCC samples and cell lines when compared with normal tissues and HK-2. (e, f) The expression of miR-101-3p in RCC cell lines and 11 paired clinical specimens (^∗^*P* < 0.05, ^∗∗^*P* < 0.01, ^∗∗∗^*P* < 0.001, and ^∗∗∗∗^*P* < 0.0001).

To detect its expression in RCC tissues, we searched the GEO dataset and identified GSE95384, which conducted miRNA expression profiling array analyses in 8 RCC tumor samples compared with matched nontumor samples. It was considerably upregulated in RCC samples (fold changes > 2, *P* value < 0.05) (Figures 1(a) and 1(b)). Based on the starBase database, we also found overexpression of miR-101-3p in RCC tissues compared with normal samples (*P* < 0.01), and Kaplan-Meier survival analysis revealed that high expression of miR-101-3p was closely associated with poor survival (*P* < 0.005) (Figure 1(c)). And it was highly expressed compared with normal tissues and HK-2 (Figure 1(d)). Additionally, we also validated its expression in RCC cell lines and clinical specimens (Figures 1(e) and 1(f)). Because OSRC-2 and A498 showed higher expressions of miR-101-3p, we chose these two cell lines for further studies.

### 3.2. Overexpressing miR-101-3p Attenuated Proliferation, Migration, and Invasion of RCC Cells

**Figure 2:**
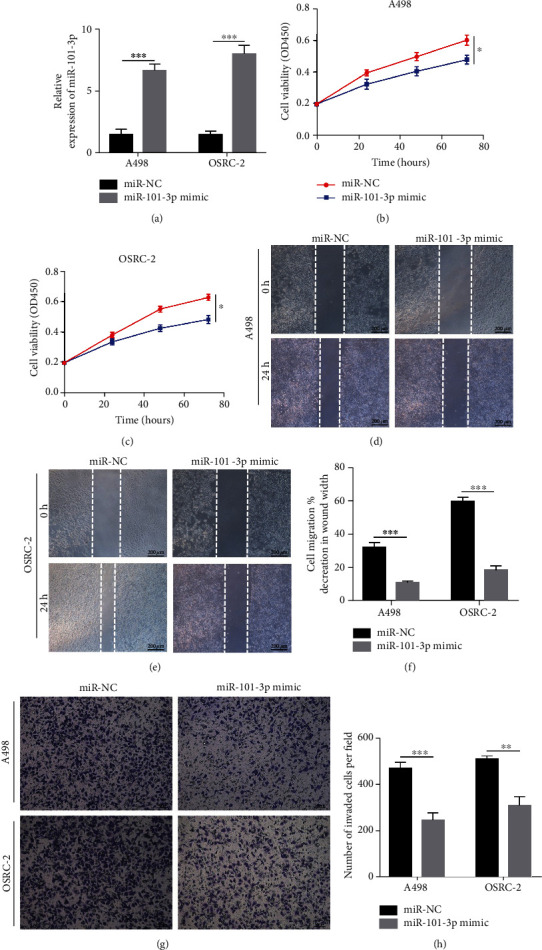
Overexpressing miR-101-3p attenuated proliferation, migration, and invasion of RCC cells. (a) miR-101-3p mimics' expression efficiency was detected by qRT-PCR in OSRC-2 and A498 cells. (b) CCK-8 assays of the viability of RCC cells after transfection with miR-101-3p mimics or NC. (c) Wound healing analysis of RCC cells after transfection with miR-101-3p mimics or NC. (d) Transwell assays of RCC cells after transfection with miR-101-3p mimics or NC (^∗^*P* < 0.05, ^∗∗^*P* < 0.01, ^∗∗∗^*P* < 0.001, and ^∗∗∗∗^*P* < 0.0001).

miR-101-3p mimics were transfected into OSRC-2 and A498 cells, and the expression efficiency was detected by qRT-PCR (Figure 2(a)). CCK-8 assays showed that the viability of RCC cells was attenuated after transfection with the mimics (Figures 2(b) and 2(c)). In addition, cell migration of OSRC-2 and A498 cells was significantly suppressed after transfection of the mimics (Figures 2(d) and 2(e)). Transwell assay also showed that overexpression of miR-101-3p mimics inhibited the migration and invasion of RCC cells (Figures 2(g) and 2(h)).

### 3.3. EZH2 Was the Downstream Target of miR-101-3p

**Figure 3:**
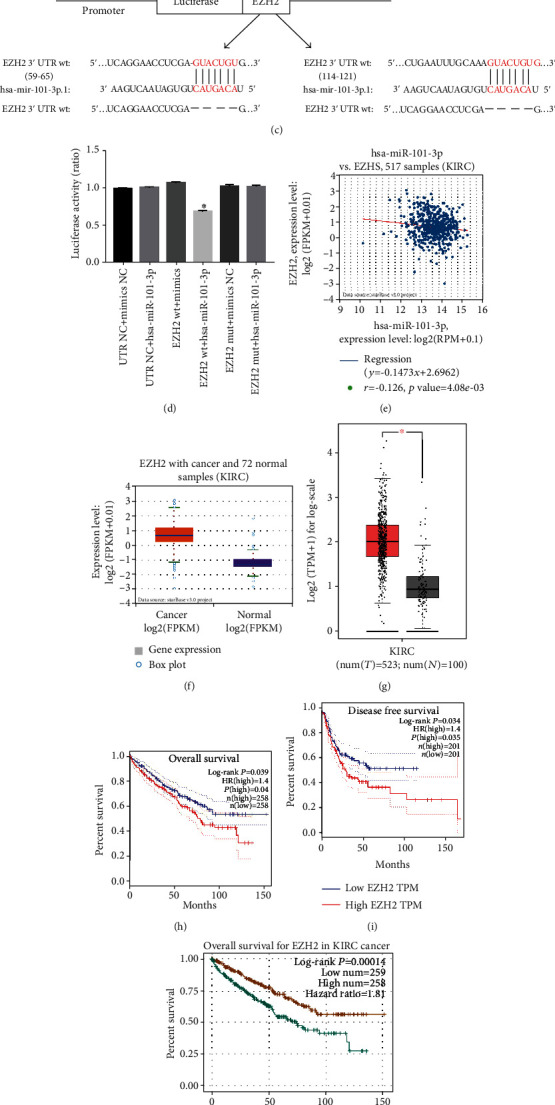
EZH2 was the downstream target of miR-101-3p. (a) The diagram of miR-101-3p potential target genes predicted by TargetScan, miRTarBase, and miRDB. (b, c) Schematic of EZH2 wild-type (WT) and mutant (Mut) luciferase reporter vectors. (d) Relative luciferase activity measured by luciferase assays in HEK293T cells cotransfected with miR-101-3p mimics or NC. (e) Pearson's correlation coefficient analysis of miR-101-3p expression levels with EZH2 in starBase. (f, g) Relative expression of EZH2 in unpaired or paired RCC tissues based on starBase and GEPIA databases. (h–j) Kaplan-Meier survival analyses of EZH2 expression with poor survival. Data indicate mean ± SD of three experiments (^∗^*P* < 0.05).

We queried the TargetScan, miRTarBase, and miRDB databases to explore downstream target genes of miR-101-3p. Seventeen candidates emerged after overlapping the results from three databases (Figure 3(a)). Since the above results show that miR-101-3p acts as an anticancer gene in RCC, we focused on tumor agonists in the predicted targets. Based on starBase and GEPIA databases, we measured the expression of RCC samples (data not shown) and found that EZH2 was apparently upregulated in RCC tissues compared with normal samples (Figures 3(f) and 3(g)). Pearson's correlation coefficient analysis also displayed that the miR-101-3p expression level was negatively correlated with EZH2 for both our clinical samples and starBase (Figure 3(e)). And Kaplan-Meier survival analyses indicated that high EZH2 expression was closely related to poor survival (*P* < 0.05) (Figures 3(h)–3(j)) to further verify that miR-101-3p targeted EZH2. We obtained the binding sites of miR-101-3p in the 3′-UTR of EZH2 and inserted the luciferase reporter into the wild-type or mutant sequence (Figures 3(b) and 3(c)). It revealed that the mimics significantly reduced relative luciferase activity in the transfected wild-type HEK293T cells compared with the mutant group (Figure 3(d)). According to interpretation, the above results imply that EZH2 is the target gene of miR-101-3p.

### 3.4. Overexpressing miR-101-3p Prevented RCC Proliferation In Vivo

**Figure 4:**
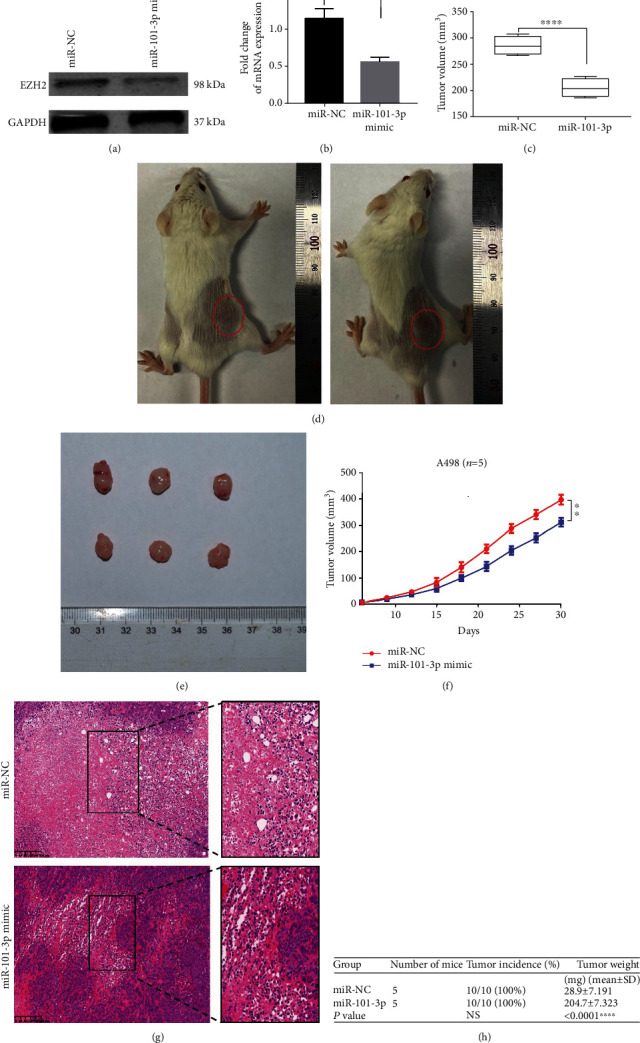
Overexpressing miR-101-3p prevented RCC proliferation in vivo. (a, b) The expression of EZH2 in A498 cell lines was measured by Western blotting and qRT-PCR. (c, d) Weights of the xenografts were shown in the box plot (*n* = 10). Macroscopic appearance of the tumors in nude mice from the 8-week-old groups. (f) Tumor volumes were periodically measured for each mouse, and tumor growth curves were plotted. Data represent mean ± SD. (g) Representative IHC staining of EZH2 from the indicated tumors (200x, 400x). (h) Tumor incidence and weights of the xenografts were shown in the table (mean ± SD) (*n* = 10) (^∗∗^*P* < 0.01, ^∗∗∗∗^*P* < 0.0001).

Western blotting and qRT-PCR verified that overexpression of miR-101-3p could reduce the protein level of EZH2 (Figures 4(a) and 4(b)). A498 cells were infected with lentivirus overexpressing miR-101-3p, and the efficacy was verified by qRT-PCR and Western blotting. We concluded that its upregulation in RCC cells obviously prevented cell proliferation (Figures 4(c) and 4(d)). Additionally, the tumor volume, weight, and growth rate of A498 cells overexpressed with it were obviously decreased, compared with those in the control group (Figures 4(e)–4(h)). Specifically, IHC analysis showed that EZH2 levels were decreased in the miR-101-3p stimulation group compared to the control group. In RCC cells, these results reported that EZH2 exerted tumor effects conversely with miR-101-3p.

### 3.5. EZH2 Restoration Reversed the Effects of miR-101-3p in RCC Cells

**Figure 5:**
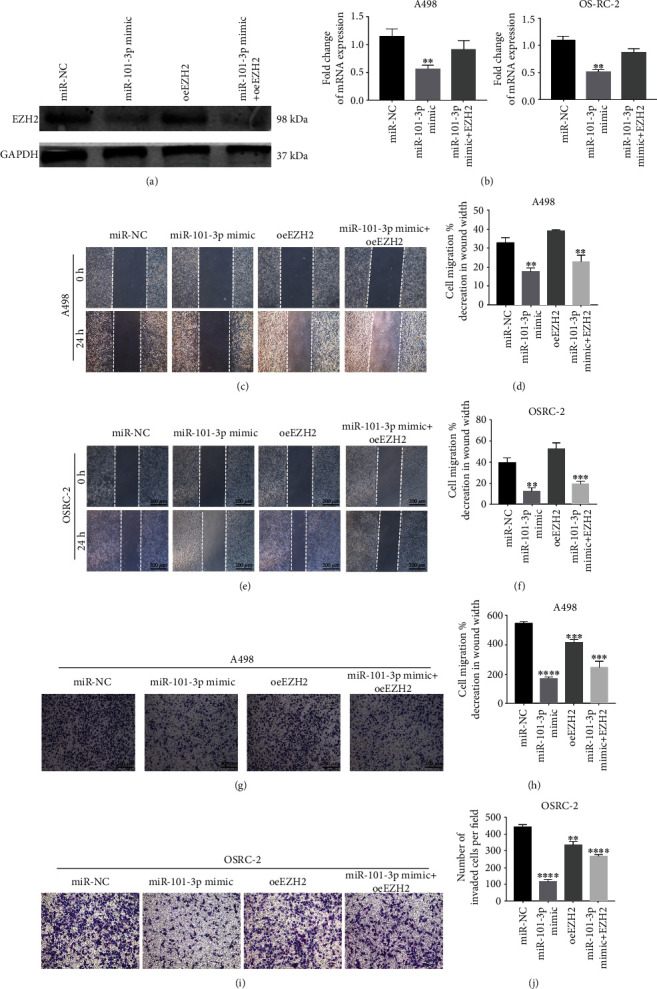
EZH2 restoration reversed the effects of miR-101-3p in RCC cells. (a, b) The protein level of EZH2 in RCC cells cotransfected with miR-101-3p mimics and EZH2 overexpressed plasmids or NC. (c–j) Wound healing and transwell assays of RCC cells cotransfected with miR-101-3p mimics and EZH2 overexpressed plasmids or NC. Data represent mean ± SD (^∗∗^*P* < 0.01, ^∗∗∗^*P* < 0.001, and ^∗∗∗∗^*P* < 0.0001).

We further validated that miR-101-3p attenuated RCC tumor characteristics via targeting EZH2 through rescue experiments. miR-101-3p mimics were cotransfected with EZH2 overexpressed plasmids or NC into RCC cells. Western blotting and qRT-PCR were implemented to assess the expression of EZH2 in RCC cells (Figures 5(a) and 5(b)). We concluded that miR-101-3p mimics inhibited the tumor characteristics of RCC cells, and EZH2 recovery partially attenuated these effects (Figures 5(c)–5(j)). And miR-101-3p mimics decreased the protein level of EZH2 while the effect was likewise reversed after transfection with EZH2 overexpressed plasmids. It suggested that miR-101-3p prevented the malignant progression of RCC by preventing the expression of EZH2.

## 4. Discussion

Currently, studies have demonstrated that miRNAs are closely related to RCC occurrence [20–23]. However, as far as we know, its expression level and function in RCC nonetheless remain to have a lot of questions. We found it was highly reflected in tumor tissues, which was consistent with data from the starBase database. Moreover, the results from miRNA expression profiling arrays (GSE16441) also revealed that it was significantly upregulated in RCC tumor specimens.

To date, miR-101-3p has been widely mentioned in other tumors; however, its role in renal carcinogenesis has not been determined. It prevents growth and metastasis of NSCLC by blocking PI3K/Akt signaling by targeting MALAT-1 [11]. Likewise, miR-101-3p prevents EMT by targeting TRIM44 to reduce glioblastoma metastasis [24]. miR-101-3p can advance the apoptosis of oral cancer cells by targeting BICC1 [8]. A recent study reported that miR-101-3p prevented retinoblastoma cell proliferation by targeting EZH2 and HDAC9 [25]. Wang and Liu concluded that autophagy in endometrial cancer cells can be prevented by miR-101-3p targeting EZH2 [26].

In this research, we observed that tumor characteristics of RCC cells were suppressed after overexpression of miR-101-3p. Additionally, Kaplan-Meier survival analyses showed that higher miR-101-3p expression indicated a better survival, which also revealed that it exerted a tumor-preventing effect on RCC. A previous study has reported that the von Hippel-Lindau (VHL) tumor suppressor could change the expression of miRNA [27]. Interestingly, we observed that miR-101-3p was relatively lowly expressed in OSRC-2 and A498 cells compared with HK-2 cells.

Luciferase reporter analysis showed that it directly targeted EZH2 by binding to 3′-UTR and inhibiting the translation of EZH2 mRNA, which was consistent with Western blotting results. Pearson's correlation coefficient analysis also disclosed that the miR-101-3p expression level was negatively correlated with EZH2 in RCC tissues. EZH2 can alter downstream target gene expression by H3K27me3 [28]. EZH2 has been well studied in prostate cancer, and its mutations are the leading cause of the progression of prostate cancer [29]. Recently, EZH2 was said to play an important role in malignancy [28, 30]. For instance, Xia et al. showed that EZH2 promotes the aggregation of macrophages and the invasion of lung cancer by enhancing the expression of CCL5 [31]. EZH2-mediated miR-139-5p regulated pancreatic cancer's epithelial-mesenchymal transition and lymph node metastasis [32]. As far as we know, the role of EZH2 in RCC has not been previously studied.

In summary, we revealed that miR-101-3p acted as an antioncogene to prevent the progression of RCC through targeting EZH2, which perhaps may provide a new therapeutic target for RCC.

## Data Availability

The related data would be provided if required.
